# Dual Effects of Non-Coding RNAs (ncRNAs) in Cancer Stem Cell Biology

**DOI:** 10.3390/ijms21186658

**Published:** 2020-09-11

**Authors:** Athina A. Kyriazi, Efstathios Papiris, Konstantinos Kitsos Kalyvianakis, George Sakellaris, Stavroula Baritaki

**Affiliations:** 1 Laboratory of Experimental Oncology, Division of Surgery, School of Medicine, University of Crete, 71500 Heraklion, Greece; bio2443@edu.biology.uoc.gr (A.A.K.); stathis_kns@outlook.com.gr (E.P.); kostis@kitsos.gr (K.K.K.); 2 Surgery Unit, University General Hospital, 71500 Heraklion (PAGNH), Greece; georgesakellaris@gmail.com

**Keywords:** cancer stem cells, long non coding RNAs, micro-RNAs, circular RNAs, cancer metastasis

## Abstract

The identification of cancer stem cells (CSCs) as initiators of carcinogenesis has revolutionized the era of cancer research and our perception for the disease treatment options. Additional CSC features, including self-renewal and migratory and invasive capabilities, have further justified these cells as putative diagnostic, prognostic, and therapeutic targets. Given the CSC plasticity, the identification of CSC-related biomarkers has been a serious burden in CSC characterization and therapeutic targeting. Over the past decades, a compelling amount of evidence has demonstrated critical regulatory functions of non-coding RNAs (ncRNAs) on the exclusive features of CSCs. We now know that ncRNAs may interfere with signaling pathways, vital for CSC phenotype maintenance, such as Notch, Wnt, and Hedgehog. Here, we discuss the multifaceted contribution of microRNAs (miRNAs), long non-coding RNAs (lncRNAs) and circular RNAs (circRNAs), as representative ncRNA classes, in sustaining the CSC-like traits, as well as the underlying molecular mechanisms of their action in various CSC types. We further discuss the use of CSC-related ncRNAs as putative biomarkers of high diagnostic, prognostic, and therapeutic value.

## 1. Introduction

Although significant progress has been made on elucidating the mechanisms of malignant cell transformation and tumor initiation, cancer itself remains a “serial” killer, counting for more than ten million deaths worldwide every year. Since the end of the last century, the so-called hierarchical model of tumorigenesis has been widely accepted and recognized by the scientific community as the prevalent one [1]. Unlike the stochastic model, which claims the equal potential of all cells contained in a tumor mass to initiate carcinogenesis, Bonnet and Dick in 1997 first reported that only CD34+/CD38– cells from patients with acute myeloid leukemia (AML) could provoke hematopoietic malignancies in immunodeficient mice [2]. Along the way, further research justified that most tumor bulks appear heterogeneous, with only a minor cell fraction, now known as cancer stem cells (CSCs), being able to induce carcinogenesis and sustain tumor heterogeneity [1,3]. According to the hierarchical model, tumor heterogeneity results from the asymmetric and symmetric division of CSCs, while the non-CSC types are more prone to death due to clonal expansion [1,4]. 

Like normal stem cells (NSCs), CSCs are also characterized by self-renewal capacity and the ability to give rise to non-stem-cell-like cancer cells. The decision of whether CSCs will retain the stem-like phenotype or will be differentiated into cancer cells is determined by various intracellular and extracellular factors, while it appears to be tissue-specific. For example, in liver tumors, the absence of Yap1, which is essential for CSC self-renewal and tissue-specific CSC fate determination, can transform CSCs into non-stem-like cancer cells. Oppositely, the overexpression of Yap1 can convert differentiated cancer cells of the liver into CSCs. This is a characteristic example, demonstrating that the multidirectional differentiation potential of CSCs conforms their plasticity and pairs together the hierarchical and stochastic models [1,4]. 

Besides the oncogenesis, we now know that CSCs are further involved in the progression and aggressiveness of the disease, the metastatic potential of the tumor, and the acquisition of tumor cell resistance to conventional chemo- and immune-therapeutics [3]. Therefore, CSCs have been recognized as a promising therapeutic target, while the identification of novel CSC-related biomarkers is gaining increasing basic and clinical interest.

Among the novel biomarkers, identified to play a crucial role in cancer pathophysiology, are the non-coding RNAs (ncRNAs) [5,6]. As their name suggests, the members of the ncRNA family have little to no protein coding ability, which is why they have been initially considered as ‘junk RNA.’ Recent advances in ncRNA research have revealed that they may act as key regulators of physiological programs in developmental and disease contexts; thus, they are of utmost importance [7,8]. ncRNAs including long non-coding RNAs (lncRNAs), microRNAs (miRNAs), and circular RNAs (circRNAs), are critical adjusters in an assortment of cellular elaboration by forming functional regulatory molecules that mediate cellular processes, including chromatin remodeling, transcription, post-transcriptional modifications, and signal transduction [6,9,10,11,12,13]. 

Although ncRNAs have been reported to participate in the regulation of various cancer cell-related properties, including aberrant proliferation, migration, and invasion, by acting either as onco-promoters or onco-suppressors, their role in CSC biology has not been clearly elucidated so far [6,11]. The aim of this review is to focus on the dual role of ncRNAs in CSC pathophysiology and the underlying mechanisms of this crosstalk. A deeper understanding of how ncRNAs may coordinate CSC properties could open new horizons in designing better therapeutic interventions for CSC elimination.

## 2. Major Types of ncRNAs Involved in Cancer Biology 

There are three major classes of ncRNAs reported to play a critical role in cancer pathogenesis. The classification has been based on their size and conformation.

MiRNAs: As their name declares, microRNAs (miRNAs) are small, linear, and single-stranded ncRNA molecules, with an average length of 22 nucleotides [13,14]. miRNAs contribute to a wide range of normal and abnormal biological processes by functioning in RNA silencing and post-transcriptional regulation of gene expression. miRNAs bind via base pairing to 3′ UTRs of mRNAs, causing their cleavage or translational repression [15,16]. In animal cells, the miRNA-coding genes are usually transcribed as long primary transcripts (pri-miRNAs), which are processed by the Drosha microprocessor complex into precursor hairpin stem-loop sequences (pre-miRNAs). These hairpins are exported from the nucleus to the cytoplasm, where the stem-loop is cleaved by the Dicer enzyme to produce a ~22 nt duplex. One strand of the duplex associates with an Argonaute (AGO) protein and this microRNA-ribonucleoprotein complex (miRNP) binds to 3′ UTRs of mRNAs. The Ago-miRNP complex then recruits other proteins, which typically mediate either the degradation or the translational repression of the mRNA [13]. The human protein-coding genes are under selective evolutionary pressure to maintain miRNA binding sites, also called miRNA response elements (MREs) [17]. 

Accumulating data demonstrate that miRNAs are further embraced in cancer biology [18]. Deregulated patterns of miRNA expression are a common feature among several cancer models affecting, either positively or negatively, cancer hallmarks, including malignant transformation, uncontrolled cell proliferation, resistance to endogenous and exogenous apoptotic stimuli, as well as induction of the epithelial to mesenchymal transition (EMT) and metastasis [18,19,20]. MiRNAs exert their suppressing actions in gene transcripts critical for the smooth operation of signaling molecular networks involved in the control of the above processes [21]. The major underlying mechanisms of miRNA dysregulation includes, but is not limited to, abnormal transcriptional regulation of miRNA-coding genes, loss or amplification of miRNA loci, and epigenetic changes in the miRNA biogenesis machinery [18]. The role of miRNAs has been further evaluated in CSC models [22]. A plethora of miRNAs have now been identified as typical molecular signatures of certain CSC stemness features and may therefore serve as novel biomarkers for CSC targeting [22,23]. 

LncRNAs: The long non-coding RNAs (lncRNAs) are one of the most abundant ncRNA families in humans, counting for more than 60,000 identified members [24,25]. LncRNA transcripts have a length greater than 200 nucleotides, show little to no evidence of protein-coding potential, while they display significant tissue specificity [26,27,28]. Most lncRNAs are located and transcribed as complex within the intergenic stretches of the genome, interwiring networks of overlapping sense and antisense transcripts that often include protein coding genes [29]. Transcriptomic sequencing using Next Generation Sequencing NGS has suggested that only a small proportion of the identified lncRNAs in humans may be actually biologically relevant [30]. Compelling evidence has demonstrated that lncRNAs play a critical role in regulating gene expression, mainly through cis-regulation or trans-regulation [31]. Although their function has been reported at all levels of gene regulation, including epigenetic, transcriptional, translational, and post-translational actions, the exact underlying mechanisms of lncRNA-mediated effects on gene regulation are still largely unknown [31,32].

The involvement of lncRNAs in the onset and pathophysiology of cancer has been recently demonstrated. Deregulated lncRNA expression patterns have been proved to significantly interfere with cancer hallmarks, including aberrant tumor cell proliferation and tumor aggressiveness, reflected by increased metastatic potential [33,34]. Therefore, lncRNAs may be considered putative therapeutic targets for cancer management.

CircRNAs: Forty years ago, a new class of ncRNA was described, now known as circular RNA (circRNA) [35]. The circRNAs have covalently closed-loop structures that are highly stable and conserved among species [36]. These RNAs are single-stranded RNAs, which are commonly generated by the pre-mRNA splicing machinery, via back-splicing reaction, in which an upstream acceptor site is joined with a donor by intronic repeat sequences that base-pair to one another and bring the intervening splice sites into close proximity [37,38]. The majority of the circRNAs are rarely produced and accumulate to low levels, although some are expressed at levels 10 fold higher than their associated linear mRNAs [39,40]. Although a large number of circRNA functions are still unknown, within the last decade, there have been compelling evidence demonstrated that circRNAs may interact with other ncRNAs such as acting as miRNA sponges or competing with pre-mRNA splicing. They can further interact with RNA-binding proteins and participate in protein translation, nuclear translocation, and scaffolding, while they can serve as autophagy regulators [36,41]. Recently, an important role of circRNAs has been identified in the pathophysiology of several diseases including cancer. A growing number of reports has demonstrated the involvement of circRNAs in oncogenesis and cancer progression by regulating tumor growth, invasion, metastasis, vascularization, and resistance to apoptosis [35]. Therefore, circRNAs have been suggested as a novel class of biomarkers and therapeutic targets in oncology. CircRNAs may further exert dual roles in the functions and properties of CSCs, a topic that we discuss in the following paragraphs. 

## 3. Normal vs. Cancer Stem Cells 

Stem cells are defined by their pluripotency to be developed into more than one specialized multipotent progenitor cell type, which, in turn, will generate the terminally differentiated and mature cell or tissue types in an organism. There are two main types of normal stem cells (NSCs) based on their pluripotent potential. The embryonic stem cells, which arise from the premature divisions of the fertilized egg and give rise to all tissue types during embryogenesis, and the somatic or adults stem cells, which exist as a “bulk stock” in all developed tissues and organs for the maintenance of tissue homeostasis, while contributing to tissue repairing [42]. Somatic stem cells can be categorized as unipotent, oligopotent, or multipotent depending on how they differentiate [43]. Scientists nowadays can create pluripotent stem cells by reprogramming somatic stem cells to act like embryonic stem cells. These reprogrammed cells can be used for reclamation of the damaged organs, meliorate organ transplant, and to test drugs that are under development [42].

Stem cells are characterized by two fundamental properties: Self-renewal and differentiation, while some stem cells can also present high multiplying potential [42]. The maintenance of a stable number of stem cells can be materialized through a single mitosis, in which at least one of the two daughters preserve stemness [44]. This type of mitosis, known as asymmetric division, is an exclusive property of stem cells [45]. Alternatively, stem cells may go through symmetric divisions generating either two stem cells [44], thus leading to an enlargement of the stem cell pool, or two non-stem-cell-like progenitors, as it happens after tissue damage that has led to the loss of differentiated cells [45]. The choice between symmetric or asymmetric divisions allows stem cells to retain themselves but also to create progenitor cells committed to give rise to highly specialized cell phenotypes necessary for the function of specific organs. Although the progenitor cells have lost the pluripotent potential of a stem cell and the related stem-like features, they are the cornerstone of organogenesis [42]. 

The reasonable questions one can address are what is different with cancer stem cells (CSCs), what is their origin, and how are they related to tumor initiation. CSCs constitute a minor cell sub-population within a tumor bulk. Compelling evidence demonstrates that CSCs may occur from normal stem cells or progenitor cells, via multiple mechanisms, including (1) fusion of an NSC with a cancer cell, (2) unceasing symmetric divisions of NSCs that may accumulate mutations and convert them into CSCs, (3) a metabolic alteration in somatic or differentiated cells that could reprogram them into CSCs, and (4) ionizing radiation, damage, or exposure to toxic substances that can transform somatic cells into CSCs [46]. Although both NSCs and CSCs may share common cell surface markers and use the same signaling pathways, these networks are often deregulated in CSCs due to genetic and epigenetic mutations that can lead to malfunction of the molecular networks and the stem cell features they control [42]. Several lines of evidence have also demonstrated that circulating cancer cells appear to share common features and properties with CSCs and, therefore, they can be considered as “soluble” CSC markers [1,4,47]. Interestingly, it has been reported that, in contrast to NSCs, CSC self-renewal ability is significantly upregulated, leading to CSC overpopulation. This uncontrollable CSC production is thought to be attributed to a higher rate of symmetric over asymmetric cell division observed in CSCs [42]. 

Along with the exclusive self-renewal ability, CSCs are also able to give rise to non-stem-cell-like cancer cells through asymmetric and symmetric divisions [42]. The tumor heterogeneity related to the different tumor-initiating potential of the cells constituting the tumor mass also reflects diverse cell responses to endogenous and exogenous apoptotic stimuli, as well as varied migratory and invasive abilities. Although the non-stem-like cancer cells and NSCs, especially those in the bone marrow, appear to be the most sensitive to anti-neoplastic regimens, in vitro and in vivo studies have demonstrated that CSCs acquire resistant phenotypes to conventional chemotherapy and endogenous immune-mediated cytotoxicity, while they are prone to give distant metastases [42]. As such, CSCs are thought to be the ones to blame for tumor regressions after therapy and for aggressive tumor phenotypes [42]. 

The special features of CSCs can be considered a result of modifications on molecular signaling pathways that control the survival and function of stem cells. Studying and understudying these changes at multiple molecular levels, as well as the underlying triggering factors, may allow a more efficient therapeutic targeting and management of malignancies.

### 3.1. Deregulated Signaling Pathways in CSCs and Their Impact on Cancer Cell Stemness

The major signaling cascades, known to designate the homeostasis of NSCs and their features, are the Notch, Hedgehog, and Wnt/β-catenin pathways [48,49]. The above cascades are an interconnected network of signaling intermediators that stoke into one another and impact the SC characteristics. Several markers mostly related to these molecular pathways are currently used to enrich cell isolates for stem cells or help in their isolation [48]. Under the pressure of accumulated mutations or loss of regulatory control, the above networks can be activated abnormally or deregulated, leading to altered cell features present in CSCs. Such abnormalities can be conducive to increased self-renewal capabilities, and cell proliferation and differentiation of CSCs [48]. 

The Notch pathway is known to regulate CSC self-renewal. Notch receptors after binding their ligands are disrupted by γ-secretase into a firm intracellular domain, which can shift to the nucleus and activate the genetic transcription of Notch targets [50]. It is worth noticing that the role of the Notch pathway in the self-renewal of CSCs depends on the presence and type of Notch receptors in specific cancer types. As an example, the self-renewal of liver CSCs is tightly regulated by the type 2 Notch receptor [51]. Accordingly, the activation of the Sonic Hedgehog (SHH) signaling, known to contribute to bladder cancer progression and other tumors, is regulated by two receptors: The Patched and Smo. The Smo receptor, after ligation, is activated, while the Patched, which suspends the activation of SHH, is exempted. As a result, Hedgehog target genes are constitutively expressed [52]. As an example, GALNT1 (a glycotransferase highly expressed by BCMab1+/CD44+ bladder CSCs) can stimulate the Hedgehog signaling through O-linked glycosylation of SHH, thus promoting CSC self-renewal in bladder cancer [52]. 

Furthermore, a third signaling cascade, known as the Wnt/β-catenin pathway, has been shown to play a vital role in normal and pathological cell stemness. This pathway is known to be activated by β-catenin, leading to expression of target genes that regulate stem cell self-renewal [53]. When Wnt signaling is deactivated, β-catenin can be found in the cytoplasm forming the adenomatous polyposis coli (APC)-degrading complex [1,54]. Contrarily, when β-catenin is activated, the APC degrading complex is disintegrated and β-catenin is transferred into the nucleus, where it interacts with TCF/LEF to form the β-catenin-activating complex [1,48,55]. Constitutive activation of Wnt/β-catenin is a hallmark of CSC pathophysiology and a putative therapeutic target for CSC elimination. 

### 3.2. Deregulated CSC Niche and Its Impact on CSC Properties

Stem cells live in a dynamic microenvironment, called niche, which provides them with essential components for their survival [56]. Depending on the needs of each tissue, the niche is able to adjust stem cell behavior by tightly regulating their self-renewal, differentiation, and quiescence [57]. In general, the niche retains stem cells in a low metabolic mode in order to obviate their exhaustion while protecting them from accumulating mutations that can result in their malignant transformation into CSCs [58]. The ability of a stem cell to seed properly in its niche is critical for its survival and functionality, while it is a prerequisite for retaining the tissue stem cell pool for long-term self-renewal [45]. 

On the other hand, CSC niches are microenvironments characterized by a constant supply of a wide range of signals capable of supporting a long-lasting CSC survival, self-renewal, and angiogenesis independent of the needs of the host tissue [4]. CSC niches may also provide a variety of factors that stimulate the invasion and metastasis of CSCs [1]. Based on the nature of these factors and the processes they induce, CSC niches can be characterized as inflammatory, perivascular, or premetastatic [1]. We now know that there is a crosstalk among niches of different characteristics. As an example, in an inflammatory niche, the tumor-associated macrophages (TAMs) and CD4+ T cells stimulate the release of TNPα, which, in turn, activates NF-κB signaling and the downstream targets Slug, Snail, and Twist, resulting in induction of the epithelial-mesenchymal transition (EMT), and CSC invasion and migration to distant locations [1]. 

Early reports demonstrated that CSC niches are mainly located near blood vessels. It was further shown that the number of CSCs is positively associated with vascular intensity, while the CSCs are in close proximity to vascular endothelial cells [59]. It is worth mentioning that when vascular endothelial cells and CSCs grew together, the latter formed oncospheres five times larger than CSCs alone, thus suggesting an interplay among CSC niche, tumor angiogenesis, and tumor growth [1]. The perivascular niche shields cancer stem cells from damage caused by radiation, while vascular endothelial cells reinforce CSC self-renewal through the VEGF-Nrp1 signaling pathway [1]. The premetastatic niche is rich in blood vessels and factors that maintain CSC survival and plasticity, along with increased metastatic potential [60]. 

Furthermore, CSC niches may be highly hypoxic. The hypoxic factor 1α (HIF1α) contributes to CSC self-renewal through activation of Notch and TGFβ signaling and production of ROS. Hypoxia also keeps CSCs in a quiescent state and decreases DNA damage, while it protects them from the destructive effects of radiation and chemotherapy [1]. 

Summarizing, it is apparent that CSCs differ significantly from NSCs not only in the “intensity” of common features and the ability to acquire new properties, but also in the homing microenvironment where they survive and maintain this special phenotype. Although the major features of both stem cell types are under the control of common molecular signaling pathways, there is now clear evidence indicating that the deregulation of these circuits in CSCs may be promoted by specific gene signatures. Many ncRNAs are now included in the list of the CSC-related gene signatures that can positively or negatively affect their pathophysiology and may, therefore, serve as putative CSC biomarkers and therapeutic targets.

## 4. Dual Effects of ncRNAs in CSC Biology 

Novel experimental findings have revealed that ncRNAs are very important adjusters in an assortment of cellular elaborations, including stem cell biology [61]. Members of ncRNAs, including mainly miRNAs and lncRNAs, have been proposed to regulate the developmental stage of stem cells [19,61,62,63]. In CSC biology, ncRNAs have been reported to operate either as oncogenes or tumor suppressors, thus exerting dual effects on CSC behavior and properties [19]. 

### 4.1. miRNAs and CSCs

A number of different miRNAs have been identified as critical regulators of cancer development and progression, through sustaining or inhibiting CSC functions [19,22]. A list of major miRNAs reported to exert dual effects on CSC properties are summarized in Table 1.

MiR-589-5p promotes the spheroid formation of CD133+ hepatocellular carcinoma (HCC) cells and their tumorigenicity in vivo by targeting the IL-6 pathway inhibitors SOCS2/5 and PTPNI/11, while it further accelerates tumor chemoresistance via regulation of Stat3 signaling [77]. High levels of miR-199a, detected in breast CSC populations, were also found vital for maintenance of the CSC self-renewal capability. The underlying mechanism involves direct targeting of a nuclear, ligand-dependent corepressor (LCOR) that sensitizes CSCs to interferon responses and subsequent differentiation or senescence [99]. In melanoma, overexpression of miR-765 by the ER-stress-induced HOXB9 protein results in downregulation of the FOXA2 transcription factor, which, in turn, de-represses the CSC self-renewal capacity and protects them from apoptotic cell death [100]. Concomitantly, the high expression of miR-126 in chronic myelogenous leukemia (CML) is associated with induction of leukemia stem cell (LSC) self-renewal. Low miR-126 levels in LSCs can be sustained by BCR-ABL-mediated phosphorylation of SPRED1, which disrupts RAN/Exp-5/renal cell carcinoma (RCC)1, a complex needed for pre-miR-126 maturation [101]. Tumor treatment with tyrosine kinase inhibitor (TKI) may inhibit BCR-ABL kinase activity, resulting in miR-126 overexpression and subsequent activation of MAPK/ERK signaling that favors LSC survival [101,102,103]. On the same context, miR-548c-3p levels have been reported elevated in a CD133^+^α2β1^Hi^ prostate cancer subpopulation, contributing to a stem-like phenotype that is characterized by increased self-renewal abilities and radioresistance [75]. However, the underlying molecular mechanism of miR-548c-3p in CSCs remains largely unknown. Likewise, miR-1246 overexpression was shown to promote stemness and drug resistance in NSCLC and pancreatic tumors, via targeting the tumor suppressors MT1G and CCNG2, respectively [73,74]. MiR-25 enhances the resistance of liver CSCs to TRAIL-mediated apoptosis by direct targeting of PTEN [65]. By contrast, miR-15a acts as a tumor suppressor by regulating the expressions of YAP1, DCLK1, BMI1, and Bcl. Its downregulation in colorectal cancer promotes stem-cell-like properties, while a miR-15a mimic has been developed as a potential therapeutic molecule to eliminate resistant colorectal CSCs [72]. 

Given that Notch signaling activation is essential for tumorigenesis and cancer aggressiveness mediated by CSCs, it has been suggested that miRNAs able to modulate this cascade may eventually interfere with CSC functions [22]. MiR-1 was among the first single miRNAs identified to directly regulate Notch, via the Dll-1 protein in mouse embryonic stem cells [107]. MiR-1 induction was further shown to reverse lung cancer cell-drug resistance [108], while it could suppress colorectal tumor development and metastasis, by regulating the activities of Notch3 and its downstream target Asef [22,64]. Underexpression, or loss, of miR-1 allows Notch3 hyperactivation and subsequent Asef overexpression, which, in turn, promotes the growth, migration, and invasion of colorectal cancer cells [22]. Notch-dependent miR-1 onco-suppressive functions have also been identified in other malignancies, including hepatocellular, lung, prostate, and head and neck carcinomas [22,109]. The oncogenic activation of Notch may be accelerated by other single miRNAs, such as miR-146a and miR-143, resulting in induction of CSC traits [22,66]. MiR-146a overexpression, frequently observed in H-Ras+ breast cancer models, facilitates the translational suppression of the Notch signaling inhibitor NUMB, thus firing up Notch and its downstream target NF-κB in pro-tumorigenic mammary stem cells [66]. Along with miR-146a, mir-143 has also been associated with the oncogenic activation of Notch1-3 pathways in different cancer types [22,110]. In lung cancer models, the dysregulation of miR-708-5p expression has been positively linked with cancer cell stemness and its related pathogenesis, while the upregulation of miR-19a and miR-19b appears to be vital for conservation of lung CSC tumorigenic activity, via constitutive activation of the Wnt/β-catenin pathway [23,67,68].

Contrarily, miR-451 was evidenced to negatively regulate Wnt signaling, which is functionally essential for the maintenance of colorectal CSC-like features, including self-renewal, tumorigenicity, and drug resistance [69,70]. Induction of miR-451 in colorectal cancer cells was able to obstruct Wnt activation by suppressing the translation of its upstream activators, macrophage migration inhibitory factor (MIF) and COX-2 [70,71]. As such, miR-451 may act as a CSC suppressor. Along with miR-451, miR-320 was also shown to negatively impact Wnt activation in prostate cancer (PCa) cells, via direct targeting of the major Wnt activator, β-catenin [70,76]. Indeed, an inverse correlation between miR-320 and β-catenin expressions has been monitored in CD44+ PCa cells, thus suggesting that miR-320 may inhibit Wnt/β-catenin-mediated downstream effects on CSC pathophysiology in prostate cancer [70]. On the same context, miR-148a downregulation, observed in a clinically aggressive stem-cell-like subtype of hepatocellular carcinoma (HCC), was associated with direct suppression of a type I receptor of Bone Morphogenetic Proteins (BMPs), namely Activin A Receptor Type 1 (ACVR1), which, in turn, results in inhibition of BMP/Wnt signaling [94]. MiR-302a/d was further identified in HCC as a suppressor of liver CSC self-renewal, by promoting cell cycle arrest via downregulation of the E2F7/AKT1/β-catenin/Cyclin D1 axis. MiR-302a/d inhibits Akt1/Cyclin D1 signaling and the nuclear levels of β-catenin by direct binding to their transcriptional activator E2F7. [95]E2F7Akt1/CyclinD1 path is known to promote cell cycle progression and proliferation, while Cyclin D1 is a downstream target of Wnt [95]. Additional miRNAs have been reported to play a regulatory role in the survival and features of hepatic CSC, by exerting dual effects on the Wnt/β-catenin signaling path. MiR-214 reduces EpCAM+ hepatic CSC numbers by direct targeting of CTNNB1-encoded β-catenin mRNA or EZH2 mRNA [104], while miR-1246 functions as an activator of the Wnt/β-catenin pathway in CD133+ liver CSCs via targeting β-catenin inhibitors Axin2 and GSK3β [105]. Downregulation of the Wnt activator Frizzled4 has also been identified as the underlying mechanism of let-7b-mediated CD24+/CD133+ hepatic CSC elimination [106]. What is noteworthy is that in relevant studies, different hepatic CSC-like subpopulations were characterized by distinct miRNA expression patterns, with some of them showing tissue specificity [111]. 

In pancreatic CSCs, overexpression of miR-17-92 inhibited their self-renewal and tumorigenic abilities, as well as the expression of stem-cell-like surface markers, by downregulation of NODAL/ACTIVIN/TGF-β signaling, which is critical for pancreatic CSC pool maintenance and stemness [96,97]. MiR-7, whose levels were found significantly downregulated in breast CSCs (BCSCs), has been associated with inhibition of BCSC proliferation, migration, and invasion to distant organs, through direct suppression of the oncogene SETDB1 and its downstream target Stat3 ([79]). Recent findings suggest that lncRNA HOTAIR might be responsible for miR-7 downregulation in BCSCs [79]. Concomitantly, miR-93 has been reported to interfere with the fate of BCSCs by regulating their proliferation and differentiation states in vitro, and their tumorigenic and metastatic potentials in vivo [80]. Depending on the cellular differentiation state, miR-93 induction can promote the Mesenchymal to Epithelial Transition (MET) in BCSCs, mediated by inhibition of TGF-β signaling and its downstream CSC regulatory targets, including Jak1, AKT3, SOX4, and STAT3, therefore leading to CSC depletion [80]. An additional major BCSC suppressor is miR-MiR-206, which represses the expression of the actin-binding protein TWF1, which, in turn, downregulates the mesenchymal lineage transcription factors MKL1 and SRF, as well as the expression of the cytokine IL-. The above factors act as positive regulators of breast cancer progression, by promoting BCSCs self-renewal, invasion, and EMT [98]. miR-193 has been identified as an onco-suppressive miRNA, partially through indirect regulation of the CSC tumorigenic potential. miR-193, when induced, is able to inhibit tumorigenicity and the invasiveness of developmentally diverse cancer cell types. By direct targeting of PLAU and K-Ras mRNAs [81], MiR-193 has been further shown to inhibit cancer progression in colon- and breast-derived xenografts [70].

Along with single miRNAs, families of miRNAs have also been reported to exert dual functions on CSC pathophysiology. MiR-34 is one representative cluster, whose members have been shown to mediate tumor-suppressing effects, including inhibition of CSC self-renewal and differentiation in colorectal, pancreatic, lung, breast, and prostate cancers [22,70]. The MiR-34 cluster consists of three members, namely miR-34a, miR-34b, and miR-34c. These members are encoded by genes located in different chromosomes, while they are associated with regulation of p53 and Notch 1/2 signaling pathways [22,70]. Low levels or loss of miR-34 cluster members have been detected in a variety of malignancies, including breast, brain, pancreatic, and non-small cell lung carcinomas [82]. In pancreatic cancer models, restoration of miR-34a expression inhibited CD44+/CD133+ CSC self-renewal capacity, through direct down-regulation of Bcl-2 and Notch signaling [22,83]. Upregulation of miR34c-3p in glioblastoma cell lines reduced the activation levels of Notch 2 and prevented cell proliferation [22,82]. Restoration of miR-34a expression in C4-2B and CWR22rv1 prostate cancer cell lines was also associated with significant inhibition of CSC self-renewal and overall tumor growth via Notch1 suppression [22,84]. 

MiR-200 is another miRNA cluster that was recently shown to be tightly involved in the regulation of CSC features in multiple tumor types, including breast, colorectal, prostate, and brain cancers [22]. The miR-200 cluster consists of five individual members, miR-200a, miR-200b, miR-200c, miR-429, and miR-141, whose overexpression results in Notch downregulation and suppression of CSC self-renewal, differentiation, and metastatic potentials in several tumor types [22,70,85,86,87,88]. The principal underlying mechanism for miR-200-mediated inhibition of Notch signaling and CSC metastasis in prostate, pancreatic, and esophageal cancers is through suspension of upstream activators of Notch, such as the connectors Notch Jagged-1 [22,70,89,90]. Moreover, low levels of miR-200c and miR-141 have been associated with increased drug resistance of pancreatic adenocarcinoma and the basal type of breast cancer, via induction of the EMT marker ZEB-ZEB-1 and, in turn, promotes the transcriptional activation of Notch, through regulation of Jagged-1 and Maml1 and 2 promoter binding activities [22,89,112]. 

MiR-200-mediated suppression of breast CSC metastatic potential has been shown to be negatively regulated by miR-22 [70,91,92]. MiR-22 acts like an epigenetic modifier in cancer, by directly targeting enzymes of the Ten Eleven Translocation (TET) family [70,93]. TET members participate in DNA demethylation, including demethylation of the miR-200 promoter [93]. As such, miR-22-mediated suppression of TET enzymes inhibits miR-200 demethylation and expression, thus promoting a CSC-related EMT phenotype, essential for metastasis [70]. 

Moreover, expression levels of miR-181 family members have also been found elevated in EpCAM+ HCCs. miR-181 contributes to maintenance of the EpCAM+ hepatic CSC stemness features and inhibition of cell differentiation by targeting the Wnt signaling inhibitor NLK and the GATA-binding protein 6 and CDX2, respectively [78].

### 4.2. lncRNAs and CSCs

Besides miRNAs, lncRNAs have also been reported to participate in the regulation of CSC- traits, including oncogenesis, metastasis, chemo/radio-resistance, and angiogenesis, in a variety of cancer types [19,113]. These functions are mainly mediated by interaction of lncRNAs with metabolic pathways like KLF4-KRT6/13, Wnt6, and PI3 kinase/CREB, which play crucial roles in maintaining CSC features [113]. A list of major lncRNAs associated with the regulation of CSC functions is summarized in Table 2.

ROR is among the recently identified lncRNAs, shown to positively affect CSC self-renewal and pool maintenance, by direct targeting of the major CSC-related transcription factors, such as SOX2, OCT4, and NANOG, and other tissue-specific transcription factors, which are necessary for the pluripotent stem cell phenotype and its local proliferation [113,114,115]. ROR has also been involved in breast cancer resistance to 5-FU and paclitaxel, while it could inflame EMT and metastasis in both breast and pancreatic tumors, via different mechanisms, including, among others, Snail and ZEB1 activation, through p53 inhibition [115,116,117,118,119,120]. CSC EMT may be further promoted by inhibition of miR-200 cluster members through ROR-mediated induction of ZEB1 [120]. 

H19 is another maternally derived estrogen-regulated lncRNA transcript proved to positively influence CSC phenotype maintenance through feedback regulation of SOX2, OCT4, and c-myc [114,121]. Abnormal expression of H19 has been associated with increased proliferation and metastasis of gallbladder, pancreatic, and breast tumors [122,123,124]. Although high levels of H19 in breast CSCs (BCSCs) appear to be critically involved in sustaining BCSC properties [125,126], there are findings indicating that neither depletion nor overexpression of H19 affect breast cancer proliferation. Therefore, it has been suggested that H19-mediated spheroid and anchorage-independent colony formation, as well as tumor-initiating functions, are not associated with CSC proliferation and self-renewal [126]. 

HOTAIR is one of the most well-studied oncogenic lncRNAs, whose overexpression has been correlated with poor prognosis and overall patients’ survival in various cancer types, including liver, colon, hepatocellular, lung, gastric, pancreatic, breast, and esophageal squamous cell carcinomas [79,113,128,151,152,153,154,155]. HOTAIR upregulation has been associated with tumor aggressiveness and increased metastatic potential [113,152,153,154,156]. Among the known HOTAIR targets are PCDHB5, ABL2, JAM2, PCDH10, SNAIL, PRG1, and laminin HOXD10 genes, all of which control oncogenic EMT and cell cycle progression [127,152,153,155,156,157,158]. In breast cancer, HOTAIR overexpression sustains the expression levels of c-myc, Twist, and miR-9, thus conferring to CSC pool maintenance and EMT promotion, while it reduces the tumor suppressor miR-7 through positive regulation of HoxD10 [79,127]. In lung cancer models, HOTAIR could activate BMI1, CD44, OCT4, and CD133, whose expression is critical for tumor cell reprogramming toward a CSC phenotype [127,128,152]. 

Furthermore, overexpression of lncARSR in primary renal tumor-initiating cells (TICs), known to contribute to tumorigenesis, progression, and drug resistance of renal cell carcinoma (RCC), has been associated with enhancement of renal TIC properties, including tumorigenicity, self-renewal, and metastasis [129]. The underlying molecular mechanism of action involves direct binding of lncARSR to YAP, which, in turn, inhibits LATS1-mediated YAP phosphorylation and YAP translocation to the nucleus. Given that high expression levels of lncARSR and YAP have been correlated with poor prognosis of RCC patients, lncARSR has been suggested as a putative prognostic biomarker in RCC [129]. 

A growing number of lncRNAs associated with regulation of Wnt-dependent CSC functions have been identified in liver malignancies [111]. Lnc-β-Catm, lnc-TIC1, lnc00210, lncSAMΜSON, lncFZD6, lncTCF7, lncDANCR, and lncCUDR are all overexpressed in liver CD133+ CSCs, accelerating their self-renewal capacity and tumorigenicity, through activation of the Wnt/β-catenin signaling. Specifically, lnc-β-Catm and lnc-TIC1 prevent β-catenin phosphorylation and degradation by direct binding to the N-terminal of β-catenin, or induction of EZH2-mediated β-catenin promoter methylation, respectively [143,144]. Alternatively, lnc00210 blocks the interaction of β-catenin with the negative regulator of Wnt activation, CTNNBIP1, by direct binding to CTNNBIP1 [145], while lncSAMΜSON facilitates CTNNBIP1 suppression via induction of EZH2-mediated methylation of CTNNBIP1 gene promoter [111,146]. B-catenin’s stability and expression is further advanced by lncDANCR and lncCUDR, which prevent miRNA-mediated degradation of β-catenin mRNAs and promote β-catenin promoter-enhancer chromatin DNA looping formation mediated by CUDR-CTCF complex, respectively [131,132]. High levels of lncCUDR, combined with high levels of Cyclin D1 or low levels of PTEN, in liver CSCs (LCSCs) have been further associated with increased LCSC malignant proliferation and promotion of tumorigenesis, via activation of TERT and C-myc [159]. Moreover, lncFZD6 promotes Wnt/β-catenin signaling by activation of FZD6 expression [147], and lncTCF7 contributes to the transcriptional activation of the Wnt-inducer TCF7 by recruiting the SWI/SNF complex to its promoter [130]. Similarly, lncAPC recruits EZH2 to the APC promoter, thus suppressing APC transcription and accelerating Wnt/β-catenin signaling activation [148]. 

The self-renewal ability of liver CSCs has been further associated with lncHDAC2 action on the Hedgehog signaling pathway. LncHDAC2 interacts with HDAC2 and allows the recruitment of NuRD complex to the gene promoter of the Hedgehog receptor PTCH1 [136]. Normally, PTCH1 expression attenuates Hedgehog signaling activation by preventing association of the Smo protein with the Gli transcription factors [160]. Therefore, transcriptional repression of PTCH1 by lncHDAC-mediated recruitment of NuRD results in the Hedgehog signaling reactivation and acceleration of LCSC self-renewal and tumor propagation [136]. In addition, expression of lncHOXA10 in liver TICs favors TIC self-renewal by promoting the transcriptional activation of HOXA10, via recruitment of the NURF chromatin remodeling complex component SNF2L, to its promoter [137,138]. 

Furthermore, lnc-DILC depletion in liver CSCs (LCSCs) resulted in LCSC expansion and HCC initiation and progression through de-repression of autocrine IL-6/Stat3 signaling by the lack of lnc-DILC binding to IL-6 promoter [149]. Contrarily, lncSOX4 expression in CD133+ liver CSCs promotes CSC self-renewal and tumorigenesis, via enhancement of Stat3-induced SOX4 expression [150]. Similarly, lncRNA TUG1 reinforces the self-renewal and tumorigenicity of glioma stem cells (GSCs) by two diverse molecular mechanisms. The first involves the sponging of miR-145, which targets essential core stemness factors, while the second engages recruitment of PRC2 into the nucleus in order to suppress the expression of differentiation-related genes [139,140]. In hematological malignancies, like mixed-lineage leukemia (MLL), lncRNA LAMP5-AS1 attenuates MLL differentiation while promoting the MLL self-renewal ability, by enhancing the enzymatic activity of the methyltransferase DOT1L, via direct binding to its active site. Subsequently, DOTIL-mediated H3K79me2/me3 methylation levels are significantly augmented, resulting in the upregulation of stemness factors such as HOXA cluster and MEIS1 [141]. Overexpression of lncGATA6 in colorectal CSCs endorses cell proliferation, metastasis, and angiogenesis, leading to tumor initiation and propagation [142]. Although the underlying mechanism is still under investigation in CSCs, in normal intestinal stem cells (ISCs), lncGATA6 appears to contribute to ISCs self-renewal and stemness, via Wnt signaling activation. Specifically, accumulation of lncGATA6 in ISC nuclei promotes NURF-mediated expression of Ehf, which, in turn, activates the expression of R-spondins (RSPOs) receptors Lg4. Given that RSPOs are Wnt pathway agonists [161], the activation of their receptors Lg4/5 by lncGATA6 results in Wnt signaling upregulation [142]. 

Contrarily, lncRNA-p21 exhibits an onco-suppressive function in colorectal cancer cells by restraining colorectal CSC-like traits, such as self-renewal and tumorigenesis, via inhibition of β-catenin signaling activity [133]. LncRNA-LBCS has been reported to hold down bladder CSC self-renewal and chemoresistance, through induction of H3K27me3 methylation of SOX2, a vital gene for CSC pool maintenance. The latter is achieved by lncRNA-LBCS-mediated formation of a protein complex between hnRNPK, a RNA- and DNA-binding protein, and methyltransferase EZH2, which binds to SOX2 promoter and suppresses the gene expression [135]. Anisomycin-induced long non-coding RNA β-site APP cleaving enzyme 1 antisense strand (lncRNA BACE1-AS) has been further involved in the suppression of ovarian CD44+/CD117+ CSCs (OCSCs) proliferative and invasive abilities in vitro and in vivo [134]. LncRNA BACE1-AS induction by anisomycin in OCSCs was significantly correlated with increased production of ΒACE1 and the toxic amyloid β (Aβ1-42), thus suggesting a putative underlying mechanism of lncRNA BACE1-AS function in OCSCs [134]. 

### 4.3. circRNAs and CSCs 

In contrast to the initial characterization of circRNAs as “wrongly spliced” transcripts, recent findings have demonstrated their critical role in the onset and progression of several diseases, including cancer [41]. circRNAs have also drawn increasing attention in the era of regulation of CSC functions and features, including self-renewal, proliferation, differentiation, apoptosis, migration, and invasion [41]. Table 3 summarizes circRNAs with reported involvement in the regulation of CSC features.

The circRNA-mediated “miRNA sponge” function is the predominant molecular mechanism by which circRNAs can exert dual effects on miRNA signaling involved in the regulation of CSC properties [41,184]. For example, circ-ITCH could inhibit CSC self-renewal and stemness in lung adenocarcinoma, by acting as a sponge for miR-214, which is known to be implicated in Wnt activation, via suppression of the Wnt-Regulatory Protein CTNNBIP1 [162]. Likewise, circPTN expression in glioma stem cells (GSCs) reinforces GSC self-renewal and expression of stemness markers, by “sponging” and degrading miR-145-5p and miR-330-5p [179]. Both miRNAs act as tumor suppressors by inhibiting cell proliferation, through targeting the oncogenic proteins SOX9 and ITGA5 [180,181]. Moreover, has_circ_0020397 was able to promote colorectal cancer cell growth and invasion by inducing TERT expression, through “sponging” TERT repressor miR-138 [41,163]. TERT is an important factor for preservation of CSC-like properties by inducing EGFR and tRNA expression in CSCs and embryonic stem cells, respectively [164,165]. CircRNA_103809 is highly expressed in bladder CSCs, where it functions as a sponge for miR-511, thus inducing CSC self-renewal, migration, and invasion [178]. Accordingly, overexpression of has_circ_0071589 in colorectal cancer and has_circ_0046701 in glioma promote carcinogenesis and tumor invasion by targeting miR-600/EZH2 and miR-142-3p/ITGB8 axes, respectively [166,167]. Given the tumorigenic and invasive activities of CSCs, it might be suggested that the above circRNAs may predominantly exert their functions in CSCs.

Furthermore, circ-UBAP2 has been associated with colorectal CSC resistance to apoptosis by operating like a sponge of miR-143. miR-143 suppression, in turn, results in upregulation of the anti-apoptotic protein Bcl-2 that facilitates CSC survival [41,168]. Contrarily, circs-7 promotes ovarian CSC apoptosis, by sustaining the cytoplasmic localization of NF-κB and allowing the upregulation of ubiquitin C-terminal hydrolase L1 (UCHL1). Degradation of BACE1 by UCHL1 results in elevated expression levels of APP and Aβ1-42 peptides, which contribute to ovarian CSC apoptosis induction [134,169].

Additional circRNAs have also been reported to exert dual effects on the migratory and invasive properties of CSCs, as well as on their differentiation status [41]. In laryngeal and prostate cancer models, expression of hg19_circ_0005033 in CD133+/CD44+ CSCs has been allied with EMT promotion and the subsequent increase in CSC migratory and invasive capabilities, via Jak2/STAT5A signaling upregulation [170,171]. Concomitantly, circ-008913 has been involved in the acquisition of the CSC-related EMT phenotype in models of arsenic-induced carcinogenesis in keratinocytes [172]. In the same and other cancer models, arsenic-mediated inhibition of circ_008913 promoted the expression of CSC surface markers and the malignant cell transformation, by attenuating circ_008913-mediated “sponging” of miR-889. which in turn targets and inactivates the tumor suppressor DAB2IP, that negatively regulates CSC-like phenotypes through modulation of CD117 and ZEB1 [172,173,174]. Moreover, hsa_circ_0005075 can block hepatocellular CSC differentiation by “sponging” miR-93, which has been previously reported to regulate the proliferation and differentiation status of breast CSCs, by negative regulation of TGF-β signaling and downstream stemness-related genes, such as AKT3, SOX4, and STAT3 [41,80,175]. An additional negative regulator of CSC phenotype in HCC is circZKSCAN. This lncRNA inactivates the RNA-binding protein (RBP) FMRP, whose role is to regulate the β-catenin binding protein, cell cycle, and apoptosis regulator 1 (CCAR1). Consequently, the activation of the Wnt/β-catenin signaling pathway is hampered, as well as the CSC stemness and metastatic potential [182].

What is noteworthy is that one circRNA can regulate one CSC-related feature by distinct molecular mechanisms. For example, circ-MALAT1 promotes CSC self-renewal in HCC, either by acting as a sponge of miR-6887-3p, which results in JAK2/STAT3 signaling activation, or by disrupting PAX5 mRNA translation, via direct binding to the ribosome and PAX5 mRNA. PAX5 is a tumor suppressor and its downregulation contributes to CSC self-renewal and tumorigenicity [183]. A compelling amount of evidence has further demonstrated the critical role of circRNAs in CSC crosstalk with their surrounding microenvironment [41]. Recently, circ_100284 was reported to be secreted by exosomes of an arsenite-transformed human hepatic epithelial cell line (L-02), transferred into normal L-02 cells and inducing the malignant transformation of the former via a circRNA_100284/miR-217/EZH2 axis [185]. Moreover, other circRNAs, such as CDR1as, have been associated with extracellular matrix (ECM) organization and protein synthesis through regulation of TGFβ signaling and ECM-receptor interaction [176]. ECM is essential for CSC survival, especially during migration and invasion [177]. The above findings indicate that circRNAs not only participate in cell–cell communication, but also exert their influence on the surrounding microenvironment, which, in turn, may affect positively or negatively the CSC properties.

### 4.4. Other ncRNAs Affecting CSC Biology 

Besides the three major ncRNA types, there are additional ncRNA classes, such as piRNAs (PIWI-interacting RNA), snoRNAs (small-nucleolar RNA), and snacRNAs (non-polyadenylated (NPA)–conserved RNA) whose contribution in cancer pathophysiology is still under investigation [19]. piRNAs are small RNAs mainly found in the germline of flies and vertebrates [186]. piRNAs are related to PIWI proteins, which are known to be involved in the regulation of germline genome stability [186]. Although the exact functions of piRNAs are largely unknown, recent findings have indicated that piRNA’s role may not be limited to germline cells, but it might be extended in the regulation of tumorigenesis through CSCs [19]. For example, piRNA-651 has been found upregulated in several types of cancers including gastric, lung, mesothelial, breast, liver, and cervical carcinomas [19], and its inhibition by antisense oligonucleotides led to cell cycle arrest at the G2/M phase and subsequent repression of cancer stem and non-stem-like cell proliferation [187]. By contrast, induction of piRNA-823 in gastric cancer cells inhibited cell proliferation and tumor growth in a xenograft mouse model [188]. 

snoRNAs are ncRNAs with intermediate size, identified mainly in eukaryotes [19]. They are located in the nucleus, where the cellular locations for the synthesis and processing of cytoplasmic ribosomal RNAs (rRNAs) are [189]. snoRNAs interact with specific proteins to form snoRNPs, which are responsible for the post-transcriptional modifications of ribosomal RNAs (rRNAs) and small nuclear RNAs (snRNA)s, via 2′-O-methylation and pseudo-uridylation [189]. Recently, a new role of snoRNAs was identified in tumorigenesis. A germline homozygous 2-bp (TT) deletion of the snoRNA U50 has been associated with U50 loss, which increases the risk for prostate tumorigenesis [19]. Frequent deletion of snoRNA U50 has also been observed in breast cancer [190,191]. Although there are still no direct reports supporting a role of snoRNA in CSC functions and properties, there is increasing evidence that snoRNAs may critically affect oncogenesis by regulating protein translation [19]. 

Similarly, the functional activity of small non-polyadenylated (NPA)–conserved RNAs (snacRNAs) in CSC biology and tumorigenesis is still a question; however, analysis of sequencing data has revealed significant differences in snacRNAs between embryonic stem cells and differentiated cells [19]. 

## 5. Diagnostic, Prognostic, and Therapeutic Implications of CSC-Related ncRNAs

The way ncRNAs relate to CSC biology and contribute to cancer development and progression make them promising biomarkers of high diagnostic, prognostic, and therapeutic significance. Notably, ncRNAs, including CSC-derived ncRNAs, may be present in biological fluids—primarily in blood and serum—either as naked tumor nucleic acid (ctNAs), or a component of secreted circulating tumor cells (CTCs), or tumor-derived extracellular vesicles like exosomes [192]. The analysis of these so-called liquid biopsies offers the great advantage of detecting and monitoring the molecular identity and makeover of a certain tumor type during the disease course in a minimally invasive and individualized approach, which, in turn, gives the opportunity of precise prognostic and therapeutic interventions [193]. The significance of ncRNA expression analysis in liquid biopsies has already been justified in the diagnosis, prognosis, and recurrence monitoring of several human cancers, including lung [194], and gastrointestinal malignancies [195,196]. Liquid biopsies are further considered to hold great potential in overcoming the limitations of tissue biopsies in identifying the molecular heterogeneity between primary and metastatic tumor sites, as well as in detecting early tumor formation, unresponsiveness to conventional therapies, and disease relapse, which might be attributed to CSC occurrence and their molecular dysregulation at the ncRNA level [195]. However, despite the numerous advantages that liquid biopsy offers, it imposes certain limitations when it comes to ncRNA analysis. For example, miRNA expression levels might be detected substantially differently between blood or serum and the solid tumor of the same patient, owing to diverse mechanisms of miRNA secretion or miRNA origin (e.g., from other tissues) [197,198]. Similarly, opposite deregulation trends have also been observed in tumor tissues and liquid biopsies for lncRNAs and circRNAs. As an example, expression analysis of UCA1 lncRNA in colorectal cancer (CRC) patients showed upregulation in tumor biopsies and downregulation in serum exosomes when compared to their normal counterparts. Given that UCA1 has an oncogenic function, the above findings suggest that the accumulation of high UCA1 levels within the tumor by limiting UCA1 secretion might be vital for tumor progression [199]. By contrast, expression of TUG1, which has been previously related to glioma stem cell self-renewal and tumorigenicity [139], was found elevated in CRC serum exosomes and significantly decreased in cancer tissue, indicating that TUG1 secretion might protect cancer cells from the onco-suppressive function of this lncRNA [199]. Considering the aforementioned restrictions, the utilization of ncRNA expression patterns in liquid biopsies as diagnostic, prognostic, and therapeutic tools is under intense investigation in large cancer patient cohorts, aiming to validate their power as biomarkers in human malignancies. Keeping this in mind, establishing essential quality criteria, such as accurate ncRNA quantification in cancer and normal tissue and liquid biopsies with advanced screening methods besides qRT-PCR, (e.g., microarray technologies for high-throughput sample analysis and deep RNA-seq), as well as careful study of the ncRNA origin and the mechanisms of their secretion into body fluids, is a prerequisite toward validation of ncRNAs as cancer biomarkers [194,200,201].

In the last decade, the idea of targeting CSC-related or, even better, CSC-specific ncRNAs in the context of CSC elimination has gained increasing interest. As an alternative to the drug-mediated targeting of ncRNAs expressed in CSCs, the transport of ncRNA analogs, or inhibitors, into the cells, in the form of synthetic nucleic acids, is widely investigated. However, the selection of the proper CSC-related ncRNA candidate target that is vital for CSC existence and function, as well as the right evaluation of the mechanism by which the transported oligonucleotide may affect a decisive biological pathway in CSCs, remains critical burdens to overcome. 

From a technical point of view, the delivery of sense or anti-sense ncRNAs into alive cells may be performed either by direct injection, use of viral vectors, or non-virus-based methods [61]. Many factors can limit the outcome of each delivery method, including the short duration of ncRNA expression due to nucleases’ activation, as in the case of direct oligonucleotide injections [61], or in vivo complications due to toxicity and activation of cellular immunity, often observed in viral deliveries [61]. The use of lipid nanoparticles, as a ncRNA delivery method, is thought to be the most effective stem cell-based therapy. Nanoparticles can provide more flexibility, achieving optimized uptake by the cell, but the lack of a suitable mechanical support compound can really limit the usage of this technique [61]. 

### 5.1. Targeting miRNAs 

The use of the best characterized ncRNA class of miRNAs as putative biomarkers and therapeutic targets in oncology, in general, and CSC biology, in particular, is of great potential. This potential is further supported by the fact that circulating miRNAs may be easily detected in blood circulation and CTCs, while they are resistant to degradation [202]. Although the diagnostic power of many circulating miRNAs has been assessed in a variety of cancers, the main focus has been mainly given in gastrointestinal malignancies [196]. Notably, isolation of miRNAs from CTCs of patients with colorectal (CRC) and breast cancers could exclude the background expression of non-cancer-related miRNAs in the bloodstream, thus detecting only CTC-specific miRNAs [203,204]. In this context, there are several technical approaches for CTC enrichment and isolation; however, CellSearch© is the only FDA-approved protocol for CTC detection that can be used as a prognostic tool in metastatic breast, prostate, and colorectal cancers [205,206]. Restrictions applied to the utilization of circulating microRNA expression in body fluids as cancer biomarkers are mainly attributed to their low disease and tissue specificity and their increased editing potential [196,207]. For example, in many cancer patients, elevation of cancer-related circulating miRNAs may not reflect relevant miRNA alterations in the cancer tissue itself, but it could associate with other co-existing conditions, such as inflammatory diseases [208]. This is especially true for the circulating forms of the oncogenic and CSC-relevant miRNAs miR-21 and miR-155, whose overexpression has been linked to both inflammation and cancer, without possibility of distinguishing the source of origin in cancer patients [196]. Likewise, alterations of the same circulating miRNAs may be detected in different human malignancies, thus indicating limited organ specificity [196]. This limitation is less prevalent in exosome-derived miRNAs, whose distributions are mainly restricted to specific tissue and cancer types [209]. Significant expression differences have also been observed between the same exosome- and plasma-derived miRNAs, with exosomal miRNA levels to hold better promise for use as a more accurate cancer biomarker [210]. 

The potential diagnostic and prognostic value of miRNA levels in oncology may be justified by the fact that deregulation of a single miRNA can modify the expression patterns of multiple downstream gene targets and, subsequently, a plethora of oncogenic and/or tumor-suppressing pathways in which they participate [19]. A modified miRNA expression motif, often observed in various cancer types, can either occur directly via mutations, deletions, or amplifications on miRNA genes, or indirectly through dysregulation of transcriptional and epigenetic regulators of miRNA expression. Cellular processes like malignant transformation, tumor invasion, and metastasis, as well as persistence of CSC stemness, may reflect the outcome of deregulated miRNAs and downstream targets linked to cell cycle, apoptosis, cell migration, EMT, and CSC self-renewal control. As such, silence of miRNAs could be a promising approach for repressing oncogenic miRNAs actions, while the use of miRNA mimics could be ideal for restoring tumor-suppressing miRNA functions [19]. 

Recent findings on the diagnostic and prognostic value of miRNA expression patterns in CSCs are very encouraging. As an example, miR-548c-3p has been suggested as a prominent diagnostic and prognostic marker in prostate cancer, given that high levels of this miRNA are correlated with poor patient survival [75,211]. MiR-548c-3p overexpression has been mainly detected in a CD133^+^α_2_β_1_^Hi^ CD44^+^ prostate cancer subpopulation, and exhibits stem cell properties [212,213], while it is associated with induction of stem cell-related genes, self-renewal, and radioresistance [75]. Accordingly, elevated levels of miR-1246, which serves as a specific CSC marker that promotes stemness and drug resistance in CD44v6^+^ CSCs-CD44v6 colorectal cancer cells [73,74,214], are also related to poor overall patient survival (OS), as well as disease-free survival (DFS) [215]. Moreover, MiR-15a suppression in colorectal cancer has also been linked with poor patient prognosis. The administration of a miR-15a mimic in mouse models, propagated with colon CSCs, significantly diminished tumor development and growth. This finding suggests the prognostic value of miR-15a in colon CSCs and the potential of its therapeutic targeting in the above cell population [72]. Low miR-15a levels in pancreatic ductal adenocarcinoma (PDAC) have also been associated with poor patient prognosis, while a miR-15a mimic has shown in vitro and in vivo a functional significance and therapeutic potential [216]. 

From a technical aspect, the elevated levels of oncogenic miRNAs in differentiated and stem-like cancer cells can be modified by several means [217]. Characteristically, we can mention the suspension of oncogenic miRNA expression by antisense oligonucleotides (ASOs), which bind to miRNA targets through base-pair complementarity. ASO function is assisted by different chemical configurations, including locked nucleic acids (LNAs), anti-miRNA oligonucleotides (AMOs), and antagomirs, which are incorporated in the skeleton of ASOs, thus increasing their stability and efficacy [218]. For example, a specific antagomir has been used to knockdown the oncogene miR-21 in breast cancer MCF-7 cells, resulting in significant inhibition of MCF-7 tumor growth in vitro and in vivo in tumor xenografts through inhibiting cell proliferation and inducing apoptosis [219]. In addition, suppression of oncogenic miRNAs can be facilitated by “miRNA sponges.” These sponges inhibit miRNA activity in cultured cancer cells and mouse models by incorporating the complementary binding sites of miRNA-targeted RNA into RNA transcripts expressed from strong promoters, thus antagonizing miRNA binding to the endogenous target [218]. For example, inhibition of miR-22 by a specific “sponge” in LM2 cells, a highly metastatic breast cancer cell line, resulted in a reduction in breast cancer metastasis to the lung [217]. 

By contrast, miRNA mimics or lentiviral vectors can be used to restore the low expression levels of tumor suppressor miRNAs that are usually detected in cancer cells [220]. For example, in gastric malignancies, a mimic of the tumor suppressor miR-34 has been shown to cause cell cycle arrest in the G1 phase and induce apoptosis by attenuating the expression of miR-34 downstream target oncogenes, including BCL2, Notch, and HMGA2 [221]. MiR-34 has also been associated with p53 negative regulation in CSCs, thus causing confusion regarding its generalized onco-suppressive activity [221]. Given the ability of miR-34 to simultaneously regulate and alter several oncogenic and onco-suppressive pathways, it makes it difficult to predict its prognostic value in cancer, as initially thought [221]. In addition, the miR-34 multi-targeting property could potentially result in side-effects in healthy tissues, including cardiovascular disease [222]. That said, it becomes clear that the process of selecting an miRNA for therapeutic targeting requires a very good characterization not only of the regulation and functions of the target in the particular disease and cell model, but also of the nature of the downstream signaling pathways it affects. 

Regarding the delivery options, the first attempts of in vivo administration of naked miRNA mimics in mice lasted only a few days, thus limiting the long-term efficacy. To overcome this limitation, lentiviral vectors modified to express miRNA sequences have been utilized in xenograft cancer models for inducing stable and long-lasting miRNA expression [19]. However, there is still a long way and many obstacles to overcome for efficient miRNA delivery as a therapeutic option to treat cancer. These restrictions mainly include (1) substandard penetration of miRNAs into tumor tissues caused by mechanical and biological barriers, (2) maintenance of the firmness and probity of miRNAs in circulation, (3) induction of miRNA-related immunotoxicity, and (4) off-target effects of miRNAs [223]. Many novel carriers are constantly evolving toward the direction of a more efficient and revolutionary application of miRNAs in cancer treatment. 

### 5.2. Targeting lncRNAs 

Although our knowledge of the way lncRNAs operate is still limited, some of their properties, including tissue-specific expression patterns, make them promising novel biomarkers for several disease models [224]. In cancer, lncRNAs undoubtfully exhibit tumor-type dependent modes of expression, which, in combination with their proven regulatory role in CSC pathophysiology, make them important research tools with potential translational impacts in the fields of oncogenesis, tumor prognosis, and therapeutic targeting [19,225]. As an example, lncRNA-DANCR is overexpressed in stem-like HCC cells; therefore, its levels may serve as a prognostic biomarker for HCC patients [132]. In same tumor model, lnc-DILC expression in CD24+/EpCAM+ hepatic CSCs connects hepatic inflammation with liver CSC expansion, with low lnc-DILC levels to be predictive of early tumor recurrence and poor patient survival rates. As such, lnc-DILC may serve not only as a putative prognostic biomarker but also as a therapeutic target in liver CSCs [149].

Altered lncRNA expression levels have also been considered easily detected diagnostic biomarkers for cancer screening, given that they are traceable in body fluids such as blood, plasma, saliva, and urine [226,227]. LncRNAs can be released in exosomes or inside apoptotic bodies in conjunction with RNA-binding proteins; thus, they are resistant to RNase degradation [227,228]. By being easily detectable, lncRNAs can also be used to predict cancer behavior before, during, or after anti-cancer therapies. LncRNAs may be further utilized as therapeutic targets because of their capacities to induce their degradation, regulate their transcription, and/or prevent their interaction with other regulatory factors [229]. 

However, there are still many challenges to overcome when lncRNAs come to immediate therapeutic targeting in human diseases, including cancer. In order to understand the potential of lncRNAs in cancer and CSC-targeted therapies, it is crucial to describe each deregulated lncRNA thoroughly, possess fully its cellular functions, and identify its role in oncogenesis, disease progression, and aggressiveness [230]. Given the enormous number of lncRNAs found deregulated in cancers, it is important to identify and characterize those with critical involvement in specific processes on a precise cancer type, subtype, or subpopulation like CSCs. This task becomes even more difficult by the facts that lncRNAs are imperfectly conserved across different species, while their structural and functional data are still limited in many normal and disease models [231]. As an example, the available data on HOTAIR and H19, two of the most well-studied lncRNAs in human malignancies, whose levels have been directly correlated with tumor stage and overall patient survival, are still in their infancy, as it relates to their regulation at multiple levels, their multifaceted actions in tumor initiation and progression, as well as their candidacy as putative biomarkers for cancer diagnosis, prognosis, and therapeutic targeting [231]. However, special attention should be paid to HOTAIR when its expression in liquid biopsy is used for diagnostic purposes, because increased HOTAIR levels in blood have been associated with different types of human malignancies that, in contrast to tissue analysis, can lead to misdiagnosis [195,232,233]. 

### 5.3. Targeting circRNAs 

CircRNAs were first described forty years ago and, since then, a number of different circRNA-dependent actions have been described on a variety of physiological and pathological conditions. Findings about their involvement in cancer-related processes associated with CSC functions, such as tumor initiation, progression, metastasis, and drug resistance, are becoming increasingly undoubting, thus suggesting their significance as putative biomarkers with prognostic, diagnostic, and therapeutic value in oncology [36,194,234]. This concept is further supported by the fact that circRNAs can be easily detected in body fluids, including blood, saliva, and urine, while they can also be found in exosomes [235,236]. The stability and high specificity of many exosomal circRNAs, such as FECR1 circRNA in Small Cell Lung Cancer (SCLC) patient-derived exosomes [237], make these molecules promising exosome-based biomarkers for early cancer detection, monitoring of cancer progression or recurrence, and for prediction of the most suitable and efficient therapeutic approaches a cancer patient can follow [36]. However, elucidation of exosomal circRNA functionality and identification of their molecular targets are still under intense investigations that are expected to provide new insights in circRNA diagnostic and prognostic power in most human malignancies. CircRNAs are also abundant in free-floating cells inside body fluids, such as platelets and erythrocytes in blood circulation and CTCs [234]. Given their tissue specificity, studies have shown a higher accumulation in platelets compared to other relevant tissues tested [234]; however their detection in whole blood could only reflect the level of circulating cells [194]. A major restriction that often applies in the utilization of circulating circRNA levels in sera/plasma as putative cancer biomarkers is the frequent lack of discrimination between cancer and non-cancer patients [238], thus reducing the actual number of circRNAs that are differentially expressed in cancer liquid biopsies. 

Overall, the regulatory role of a plethora of different ncRNAs in critical cancer-related processes, including CSC-dependent oncogenesis and tumor aggressiveness, is an indisputable fact. An efficient identification and characterization of ncRNAs with CSC-related or, even better, CSC-specific deregulated expression motifs, along with a deep understanding of their action in CSC properties, might be the golden key for designing revolutionary therapeutic protocols against CSCs.

## Figures and Tables

**Table 1:** Dual roles of microRNAs (miRNAs) in the regulation of cancer stem cell (CSC)-related features.

miRNA	CSC Origin *	Direct/Indirect Targets	Effects on CSC Properties		Ref. No.
			Induction	Suppression	
miR-1	COAD	Notch3/Asef		Growth Migration Invasion	[22,64]
miR-25	LIHC	PTEN/PI3K/Akt/Bad	Resistance to TRAIL-induced apoptosis		[65]
miR-146a	BRCA	Notch/NF-κB	Self-renewal		[66]
miR-143	PCPG	Notch 1-3	Self-renewal		[22]
miR-708-5p	LUAD LUSC	Wnt/β-catenin		Stemness and related pathogenesis	[23,67]
miR-19a miR-19b	LUAD	Wnt/β-catenin	Tumorigenicity		[23,68]
miR-451	COAD	Wnt		Self-renewal Tumorigenicity Drug resistance	[69,70,71]
miR-15a	COAD	YAP1, DCLK1, BMI1, Bcl2		Stemness	[72]
miR-1246	^@^ NSCLC PAAD	MT1G CCNG2	Stemness Drug resistance		[73,74]
miR-548c-3p	PRAD	unknown	Self-renewal Radioresistance		[75]
miR-320	PRAD	Wnt/β-catenin		Self-renewal Stemness	[70,76]
miR-589-5p	LIHC	SOCS2/5/IL-6 PTPNI/11/IL-6 Stat3	Spheroid formation Tumorigenesis Chemoresistance		[77]
miR-181	LIHC	NLK/Wnt CDX2 GATA-binding protein 6	Stemness	Differentiation	[78]
miR-7	BRCA	SETDB1/STAT3		Proliferation Migration Invasion	[79]
miR-93	BRCA	TGF-β/Jak1/AKT3/SOX4/STAT3	MET CSC depletion		[80]
miR-193	BRCA COAD	PLAU K-Ras		Tumorigenicity Invasion	[70,81]
miR-34 cluster	COAD PAAD LUAD BRCA PRAD	p53 Notch1/2		Self-renewal Differentiation	[22,70,82]
miR-34a	PAAD PRAD	Bcl-2 Notch		Self-renewal	[22,83,84,85]
miR-34c-3p	GBM	Notch 2		Proliferation	[22,82]
miR-200 cluster	BRCA COAD	Notch		Self-renewal Differentiation Metastasis	[70,85,86,87]
miR-200c miR-141	PAAD BRCA	Notch		Drug resistance EMT	[22,88,89,90]
miR-22	BRCA	TET enzymes/miR-200	EMT Metastasis		[70,91,92,93]
miR-148a	LIHC	BMP/Wnt		Self-renewal Stemness	[94]
miR-302a/d	LIHC	E2F7/Akt1/β- catenin/Cyclin D		Self-renewal Proliferation	[95]
miR-17-92	PAAD	Nodal/Activin/TGF-β		Self-renewal Stemness	[96,97]
miR-206	BRCA	TWF1/MLK1-SRF/IL-11		Self-renewal Invasion EMT	[98]
miR-199a	BRCA	LCOR/IFN	Self-renewal		[99]
miR-765	SKCM	FOXA2	Self-renewal		[100]
miR-126	LCML	MAPK/ERK	Survival Self-renewal		[101,102,103]
miR-214	LIHC	CTNNB1/Wnt EZH2/Wnt		CSC number	[104]
miR-1246	LIHC	Axin2/β-catenin GSK3β/β-catenin	Stemness		[105]
Let-7b	LIHC	Frizzled4/Wnt		CSC number	[106]

* NCI-based TCGA-based abbreviations (https://gdc.cancer.gov/resources-tcga-users/tcga-code-tables/tcga-study-abbreviations): COAD, Colon Adenocarcinoma; BRCA, breast cancer; PCPG, Pheochromocytoma and Paraganglioma; LUAD, Lung Adenocarcinoma; LUSC, Lung squamous cell carcinoma; PRAD, Prostate Adenocarcinoma; PAAD, Pancreatic adenocarcinoma; GBM, Glioblastoma Multiform; LIHC, Liver Hepatocellular Carcinoma; SKCM, Skin Cutaneous Melanoma, LCML, Chronic Myelogenous Leukemia. ^@^ NSCLC: Non small cell lung cancer.

**Table 2:** Dual roles of long non-coding RNAs (lncRNAs) in the regulation of CSC-related features.

lncRNA	CSC Origin *	Direct/Indirect Target	Effects on CSC-Related Features		Ref. No.
			Induction	Suppression	
ROR	BRCA PAAD	SOX2 OCT4 NANOG Snail ZEB1	Self-renewal Drug resistance EMT Metastasis		[114,115,116,117,118,119,120]
H19	Gall- bladder cancer PAAD BRCA	SOX2 OCT4 c-myc	Self-renewal Proliferation Metastasis		[114,121,122,123,124,125,126]
HOTAIR	BRCA	c-myc Twist miR-9 miR-7	Proliferation EMT		[79,127]
HOTAIR	LUAD	BMI1 CD44 OCT4 CD133	Self-renewal Stemness		[127,128]
lncARSR	KIRC	YAP	Self-renewal Tumorigenicity Metastasis		[129]
lncTCF7	LIHC	Wnt	Self-renewal Tumor dissemination		[130]
lncCUDR	LIHC	TERT c-myc Wnt/β-catenin	Proliferation Self-renewal Tumorigenesis		[131,132]
lncRNA-p21	COAD	β-catenin		Self-renewal Tumorigenicity	[133]
lncRNA-BACE1-AS	Ovarian cancer	BACE1/Aβ1-42		Proliferation Invasion	[134]
lncRNA-LBCS	BLCA	SOX2		Self-renewal Chemoresistance	[135]
lncHDAC2	LIHC	Hedgehog	Self-renewal Tumor propagation		[136]
lncHOXA10	LIHC	HOXA10	Self-renewal		[137,138]
TUG1	GBM	miR-145 PRC2	Self-renewal Tumorigenicity		[139,140]
LAMP5-AS1	Mixed- Lineage Leukemia	HOXA cluster MEIS1	Self-renewal Differentiation		[141]
lncGATA6	COAD	Wnt	Proliferation Metastasis Tumor initiation		[142]
lnc-β-catm lnc-TIC1 lnc00210 lncSAMΜSON lncDANCR lncFZD6 lncAPC	LIHC	Wnt/β-catenin	Self-renewal	Differentiation	[130,131,132,143,144,145,146,147,148]
Lnc-DILC	LIHC	IL-6/Stat3 TNF-α/NF-κΒ		Proliferation Tumorigenicity	[149]
lncSOX4	LIHC		Self-renewal Tumorigenicity		[150]

* NCI-based TCGA-based abbreviations (https://gdc.cancer.gov/resources-tcga-users/tcga-code-tables/tcga-study-abbreviations): BRCA, breast cancer; PAAD, Pancreatic adenocarcinoma; LUAD, Lung Adenocarcinoma; KIRC, Kidney renal clear cell carcinoma; LIHC, Liver Hepatocellular Carcinoma; COAD, Colon Adenocarcinoma; BLCA, Bladder Urothelial Carcinoma; GBM, Glioblastoma Multiform.

**Table 3:** Dual roles of circular RNAs (circRNAs) in the regulation of CSC-related features.

circRNA	CSC Origin *	Direct/Indirect Targets	Effects on CSC Properties		Ref No.
			Induction	Suppression	
circ- ITCH	LUAD	^#^ miR-214/Wnt		Self-renewal Stemness markers	[162]
has_circ_ 0020397	COAD	^#^ miR-138/TERT	Growth, Invasion		[41,163,164,165]
has_circ_ 0071589	COAD	^#^ miR-600/EZH2	Carcinogenesis, Invasion		[166]
has_circ_0046701	GBM	^#^ miR-142-3p/ITGB8	Carcinogenesis Invasion		[167]
circ_UBAP2	COAD	^#^ miR-143/Bcl-2	Survival Apoptosis resistance		[41,168]
circs-7	Ovarian cancer	NF-κB/UCHL1/BACE1, APP, Aβ1-42	Apoptosis		[134,169]
hg19_circ_0005033	Laryngeal cancer PRAD	Jak2/STAT5A	EMT Migration Invasion		[170,171]
circ_008913	^&^ Skin cancer	^#^ miR-889/DAB2IP/CD117 /ZEB1	EMT Stemness markers		[172,173,174]
hsa_circ_0005075	LIHC	^#^ miR-93/TGF-β ^#^ miR-93/AKT3, SOX4, STAT3		Differentiation	[41,80,175]
CDR1as	GBM NRBL SARC SECR BRCA SKCM	TGF-β ECM-receptor interaction	Survival Migration Invasion		[176,177]
circRNA_103809	BLCA	^#^ miR-511	Self-renewal Migration Invasion		[178]
circPTN	GBM	^#^ miR-145-5p/SOX9 ^#^ miR-330-5p/ITGA5	Self-renewal Stemness markers		[179,180,181]
circ-ZKSCAN1	LIHC	FMRP/CCAR1/Wnt/ β-catenin		Stemness markers Metastasis	[182]
circ-MALAT1	LIHC	^#^ miR-6887-3p/JAK2/STAT3 ^#^ miR-6887-3p/PAX5	Self-renewal Tumorigenicity		[183]

**^#^**CircRNA acts as sponge for the indicated miRNA; * NCI-based TCGA-based abbreviations (https://gdc.cancer.gov/resources-tcga-users/tcga-code-tables/tcga-study-abbreviations): LUAD, Lung Adenocarcinoma; COAD, Colon Adenocarcinoma; GBM, Glioblastoma Multiform; LIHC, Liver Hepatocellular Carcinoma; PRAD, Prostate Adenocarcinoma; NRBL, neuroblastoma; SARC, sarcoma; SECR, secretory cancer; BRCA, breast cancer; SKCM, Skin Cutaneous Melanoma; BLCA, Bladder Urothelial Carcinoma; **^&^** Models of arsenic-induced carcinogenesis in keratinocytes.
